# Transaxillary Robotic Thyroidectomy: A Novel Technique and Update

**DOI:** 10.3390/jcm15041372

**Published:** 2026-02-09

**Authors:** Barbara Mullineris, Alice Francescato, Giovanni Colli, Davide Gozzo, Silvia Traficante, Micaela Piccoli

**Affiliations:** Department of General, Emergency Surgery and New Technologies, Baggiovara General Hospital Azienda Ospedaliero Universitaria di Modena, Via Pietro Giardini 1355, 41126 Modena, Italy; mullineris.barbara@aou.mo.it (B.M.); colli.giovanni@aou.mo.it (G.C.); gozzo.davide@aou.mo.it (D.G.); traficante.silvia@aou.mo.it (S.T.); piccoli.micaela@aou.mo.it (M.P.)

**Keywords:** thyroid, robotic surgery, remote-access surgery, minimally invasive surgery, swing technique, hybrid technique, Modena Robotic Probe, transaxillary access

## Abstract

Gasless Transaxillary Robotic Thyroidectomy (G-TART) has undergone significant refinement through the adoption of novel strategies to enhance surgical precision and safety. In this paper, we describe a novel technique that integrates dynamic endoscope repositioning, called the “swing technique”, with the use of a specialized intraoperative neuromonitoring (IONM) probe—Modena Robotic Probe—designed for robotic applications. The procedure, performed using the Da Vinci Xi system (Intuitive Surgical, Sunnyvale, CA, USA), incorporates intermittent IONM during recurrent laryngeal nerve (RLN) dissection. The swing technique involves real-time adjustment of the 30° endoscope between robotic ports to improve visualization within the confined transaxillary (TA) surgical field, particularly during contralateral dissection. Simultaneously, the Modena Robotic Probe, a custom monopolar stimulation probe developed in collaboration with Dr. Langer Medical GmbH for connection to the AVALANCHE^®^ SI2 neuromonitor, allows precise RLN mapping and verification throughout the operation. This approach could facilitate accurate anatomical tracking, minimize the risk of thermal or mechanical nerve injury, and enable safe navigation in a narrow operative TA tunnel. The adoption of advanced imaging techniques in conjunction with specialized robotic instrumentation may contribute to enhanced surgical safety and accuracy, emphasizing the importance of procedure-specific robotic approaches in thyroid surgery.

## 1. Introduction

Remote thyroid surgery represents a significant innovation in endocrine surgery, aiming to enhance surgical precision and improve patient satisfaction while minimizing the morbidity associated with conventional open thyroidectomy (OT). Among the various remote approaches developed, the gasless transaxillary (G-TA) robotic technique was the first to gain prominence [1] due to its favorable cosmetic outcomes, reproducible surgical steps, and consistently validated oncologic and surgical efficacy [2,3].

Initially developed in South Korea, the G-TA approach is now adopted in select high-volume centers worldwide. This technique enables thyroid resection through a unilateral axillary subcutaneous tunnel extending to the anterior neck, thereby avoiding a visible cervical incision. It is particularly advantageous for patients with aesthetic concerns or a predisposition to hypertrophic or keloid scarring. The approach ensures oncologic safety and surgical efficacy, particularly in institutions proficient in both robotic and endocrine surgery [4].

The introduction of the Da Vinci Xi (Intuitive Surgical, Sunnyvale, CA, USA) robotic platform in 2014 markedly enhanced the G-TA technique by offering greater instrument dexterity, high-definition three-dimensional visualization and improved ergonomics. These advancements facilitate total thyroidectomy through a single axillary incision. The Xi system’s modular configuration and simplified docking process further support complex dissection within the constrained anatomical space of the anterior neck.

A recognized limitation of the TA approach lies in the restricted visualization of the contralateral thyroid lobe during total thyroidectomy, potentially hindering the identification and preservation of the contralateral recurrent laryngeal nerve (RLN) and parathyroid glands. To address this issue, we incorporated the “swing technique” using the Da Vinci Xi system (Intuitive Surgical, Sunnyvale, CA, USA), which enhances and magnifies visualization of the contralateral lobe. This is achieved through a dynamic repositioning of robotic instruments and the 3D camera. Intraoperative nerve monitoring (IONM) through the Modena Robotic Probe supports accurate RLN identification, contributing to optimized visual clarity, surgical accuracy and safety.

Although previous publications have described the conventional technique used by our group [4,5], this refined technical report provides a detailed account of the procedural steps and operative strategies of robotic gasless thyroidectomy via a transaxillary approach performed with the da Vinci Xi platform (Intuitive Surgical, Sunnyvale, CA, USA). In this iteratively evolved technique, the swing approach and the Modena Robotic Probe were progressively integrated as technical refinements to enhance exposure of the recurrent laryngeal nerve and facilitate safe intraoperative neuromonitoring, with the potential to improve overall procedural safety, incorporating also the most recent evidence from the literature.

## 2. Gasless Transaxillary Robotic Thyroidectomy: Surgical Technique

### 2.1. Operating Room Setup and Patient Positioning

Operating room configuration is determined by the laterality of the targeted thyroid lobe in hemithyroidectomy or the dominant side nodule in total thyroidectomy. The following description outlines the standard setup for a right-sided approach. For the left side, the positioning is mirrored. Surgical equipment used in this procedure is listed in Table 1.

The patient is placed supine on the operating table with the lower limbs adducted. The head is supported by a silicone donut cushion, while a soft semicylindrical silicone pillow is placed under the shoulders to achieve gentle cervical extension. The ipsilateral arm is abducted and flexed to approximately 90°, positioned overhead to minimize the distance between the axilla and thyroid lodge, and secured on an armrest fixed to the surgical table. The contralateral arm remains adducted alongside the body. To prevent brachial plexus injury and reduce postoperative paresthesias, the arm position is verified while the patient is awake. A stabilizing cushion is positioned contralaterally to the incision site at head level to counteract the pull of the retractor, which is anchored to the surgical table during working space creation.

The surgical team comprises three members: a primary surgeon at the robotic console, and two operating surgeons, an assistant and a trainee, positioned at the patient’s right side for right-sided access. If a dual console system is available, it may be employed to promote hands-on training and accelerate the learning curve.

The operating table is centrally located within the surgical suite. The robotic vision cart is positioned near the patient’s left foot, while the patient-side cart with robotic arms is positioned near the patient’s left shoulder. The scrub nurse stands to the right of the trainee surgeon, adjacent to the Mayo stand.

The Modena Retractor (CEATEC^®^ Medizintechnik), secured contralateral to the axillary incision, facilitates creation of a gasless subcutaneous tunnel, commonly referred to as the working space, used to introduce robotic instruments. This tunnel follows subplatysmal and embryologically defined planes, extending from the axilla to the central neck. This approach minimizes postoperative adhesions and facilitates re-intervention in case of bleeding. The retractor also minimizes fogging and provides a stable operative field.

**Figure 1:**
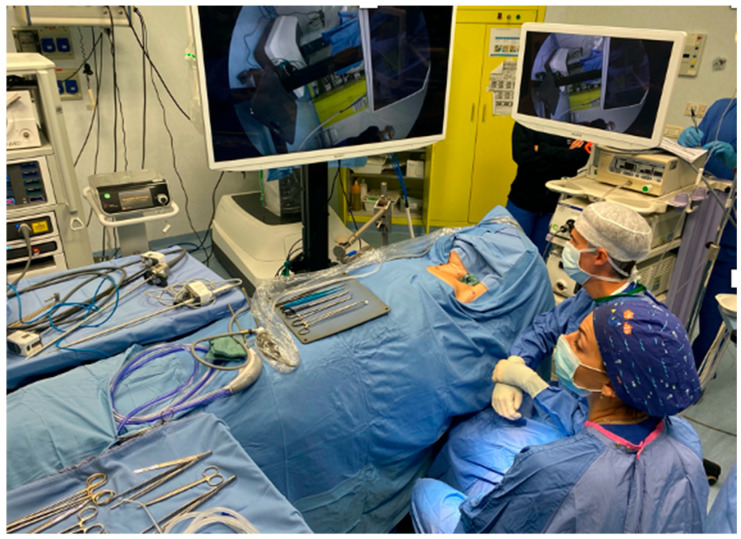
Operating room setup in hybrid technique for working space creation.

During the creation of the working space, a laparoscopic tower is positioned contralateral to the incision. A hybrid technique is employed, incorporating laparoscopic visualization to improve the precision of the dissection, reduce blood loss, and enhance flap elevation through magnified imaging. This strategy leverages the combined strengths of laparoscopic and robotic expertise (Figure 1).

Robotic transaxillary thyroidectomy is conventionally divided into three operative stages: working space creation, docking time and console time.

### 2.2. Working Space Creation

The initial phase is dedicated to the creation of a subcutaneous tunnel granting access to the thyroid compartment and employs laparoscopic expertise. For a right-sided approach, a 5 cm axillary incision is performed along the lateral margin of the pectoralis major muscle. Following identification, the muscle is dissected along its anterior fascial plane. To facilitate optimal exposure and tissue elevation, the Modena Retractor (CEATEC^®^ Medizintechnik) is positioned contralateral to the incision. An appropriate retractor blade is utilized to elevate the skin and subcutaneous tissues. A 30° laparoscopic endoscope is introduced centrally through the incision. Dissection proceeds using a Johannes forceps in the surgeon’s left hand and a laparoscopic hook in the right. The anatomical line follows the fascia of the pectoralis major muscle until the distal portion of the sternocleidomastoid (SCM) muscle becomes visible. The sternal and clavicular heads of the SCM are then separated through an avascular plane. The sternal head is subsequently elevated using the retractor blade, allowing for subplatysmal tunneling and access to the thyroid compartment.

Superiorly, the omohyoid muscle is identified, serving as the cranial boundary of the surgical field. The muscle is cranially retracted to expose the intermuscular plane between the sternothyroid muscle and the internal jugular vein. The sternothyroid and thyrohyoid muscles are lifted en bloc using the Modena Retractor (CEATEC^®^ Medizintechnik), thereby revealing the thyroid gland.

The prethyroid muscles on the right side are dissected and mobilized medially toward the left until the contralateral prethyroid muscles are reached and elevated as well with the Modena retractor blade. At this point, a longer retractor blade is substituted to maximize the working space. The thyroid is then dissected away from the overlying musculature by elevating the retractor in a caudo-cranial direction, completing the exposure for robotic docking.

### 2.3. Docking Time

Following creation of the gasless working space using the Modena Retractor (CEATEC^®^ Medizintechnik), robotic trocar placement is performed under direct vision to optimize instrument alignment and minimize tissue trauma. Through a single axillary incision, robotic trocars are introduced in a linear configuration along the anterior border of the pectoralis major muscle, which ensures optimal triangulation while preserving ergonomic instrument movement.

For a total thyroidectomy, four 8 mm robotic trocars are employed. Three are introduced through the main axillary incision, while the fourth is placed at the most caudal aspect of the incision and it is used for postoperative drain placement. In the context of TA lobectomy for benign nodules measuring less than 3 cm in diameter, a three-trocar configuration may be adopted.

**Figure 2:**
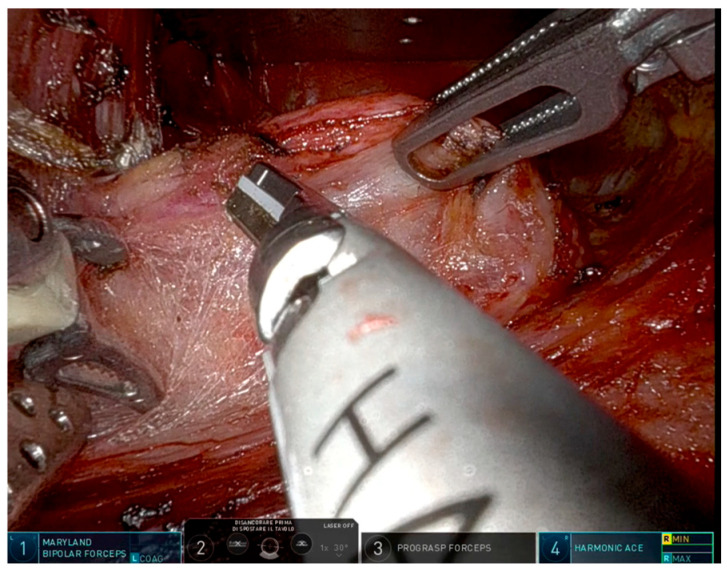
Robotic arm configuration in right superior thyroid lobe dissection.

Robotic arm configuration is as follows: a Maryland bipolar forceps is secured on arm 1 (left of the endoscope), an Ultracision© Ethicon dissector on arm 4 (right of the endoscope), and a Prograsp forceps on arm 3 (Figure 2).

Following docking, the procedure is typically managed by a single experienced bedside assistant, who is responsible for manipulating the robotic arms, exchanging instruments, and maintaining proper alignment. A modified suction retractor, characterized by its concave shape, is used to facilitate access to the surgical field. The bed-side assistant may also insert gauze, retrieve specimens, and perform aspiration with a laparoscopic suction device.

During the procedure, intermittent IONM of the RLN is conducted, introducing the Modena Robotic Probe into the working space. To improve access to the contralateral thyroid lobe, the conventional probe was modified to be thinner, longer, and concave in shape, allowing for enhanced maneuverability within the restricted operative field.

### 2.4. Console Time

The console phase is performed by a single, experienced surgeon. When a dual-console system is available, a trainee surgeon may participate, executing selected steps of the procedure under direct supervision. During this stage, robotic instruments are controlled from the console, while laparoscopic instruments are managed by the bedside assistant.

The excision of the thyroid gland is carried out during the console phase. The procedure begins with the identification and transection of the superior thyroid pole, utilizing the Maryland bipolar forceps on arm 1, a 30° robotic endoscope on arm 2, the ProGrasp forceps on arm 3, and the Ultracision© Ethicon dissector on arm 4. At this point, the superior parathyroid gland is identified and preserved.

Subsequently, the right RLN is visualized and preserved. The Ultracision© dissector and the Maryland bipolar forceps are repositioned to arm 1 and to arm 4, respectively; while the endoscope and Prograsp forceps remain on arms 2 and 3. In this configuration, the Maryland bipolar forceps is used as a true dissector, emulating the technique used in OT. The Ultracision© device, introduced from the left, ensures a safe dissection by orienting the protected portion of the blade toward the RLN trajectory.

**Figure 3:**
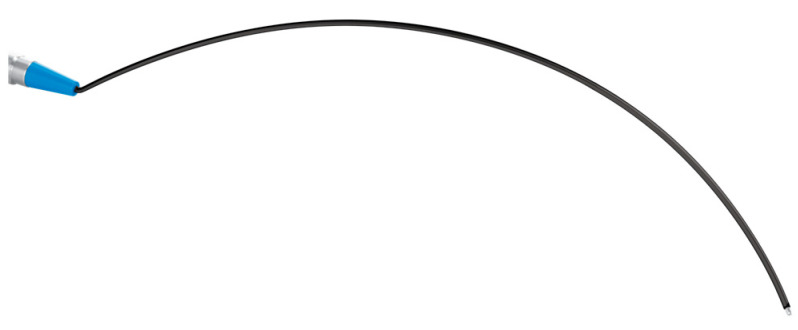
The Modena Robotic Probe.

**Figure 4:**
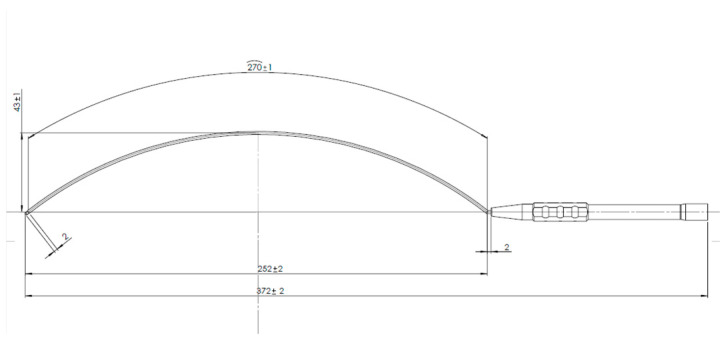
The Modena Robotic Probe project.

Intermittent IONM is performed using the Modena Robotic Probe, designed with a concave surface to facilitate use in robotic thyroidectomy. This probe enables nerve mapping and real-time verification of nerve function during dissection and following gland excision. The monopolar stimulation probe used (Model 40-0038, Dr. Langer Medical GmbH, Waldkirch, Germany) is specifically designed for use in robot-assisted surgical procedures requiring IONM (for example: transaxillary robotic thyroidectomy, transaxillary latero-cervical lymph node dissection or transaxillary Zenker’s diverticulectomy) and developed in collaboration with Dr. Langer Medical GmbH for connection to the AVALANCHE^®^ SI2 neuromonitor. The probe has a total working length of 250 mm and a curved shaft to facilitate access to deep anatomical regions within the confined operative field of the thyroid lodge. The shaft is fully isolated with polytetrafluoroethylene (PTFE) to prevent unintended electrical dispersion. The distal tip, with a diameter of 1.5 mm, is composed of biocompatible stainless steel (EN 1.4301) and serves as the stimulation interface with RLNs. The handle is manufactured from stainless steel (EN 1.4305) and terminates proximally in a blue Teca PEEK MT cone, allowing secure connection to compatible monopolar stimulation cables from the same manufacturer (Dr. Langer Medical GmbH, Waldkirch, Germany) (Figure 3 and Figure 4).

Dissection of the inferior pole is completed after identification and preservation of the inferior parathyroid gland. The retro-neural arterial branch is usually transected using the Ultracision© dissector. The thyroid gland is then mobilized from the tracheal plane using the Ultracision© dissector, with careful orientation of the isolated blade toward the trachea to prevent thermal damage or perforation. In hemithyroidectomy cases, the lobe and isthmus are typically resected and extracted through the axillary incision to expand the operative field, facilitating contralateral dissection when a total thyroidectomy is planned. If the excised lobe is small, the contralateral dissection may proceed directly without prior removal. It is also possible to use a collection bag to prevent accidental dissemination of thyroid tissue along the TA tunnel.

The next step involves the identification of the left superior thyroid pole. Instrument configuration remains unchanged: the Maryland bipolar forceps on arm 1 retract the upper pole downward and laterally, the Prograsp forceps on arm 3 elevate and medially displace the contralateral prethyroid musculature, and a specialized suction retractor, operated by the bedside assistant, mobilizes the trachea inferiorly and laterally to aid resection.

**Figure 5:**
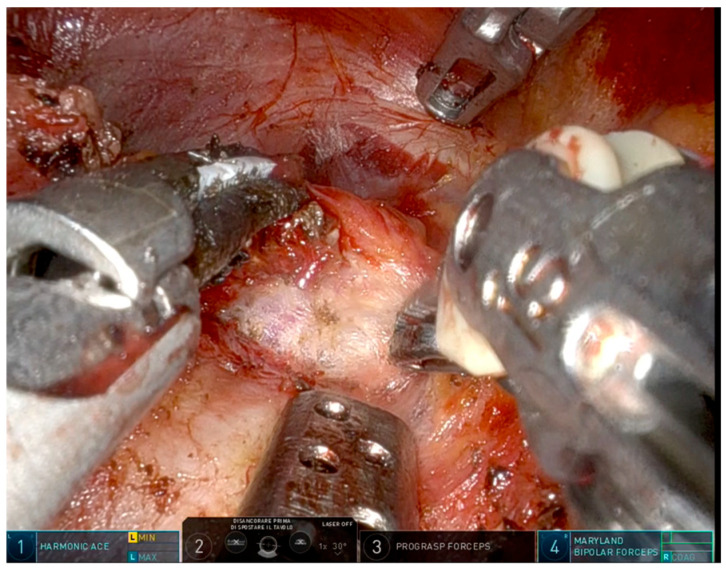
Robotic arm configuration in contralateral RLN preservation.

Dissection then continues toward the left inferior pole. The left RLN, typically closely associated with the trachea, is identified in a medial-to-lateral direction. For this maneuver, the Maryland bipolar forceps are transferred to arm 4 for optimal nerve visualization; the 30° endoscope remains on arm 2, the Prograsp forceps on arm 3 retract the thyroid upward and medially, and the Ultracision© dissector on arm 1 performs the dissection (Figure 5).

**Figure 6:**
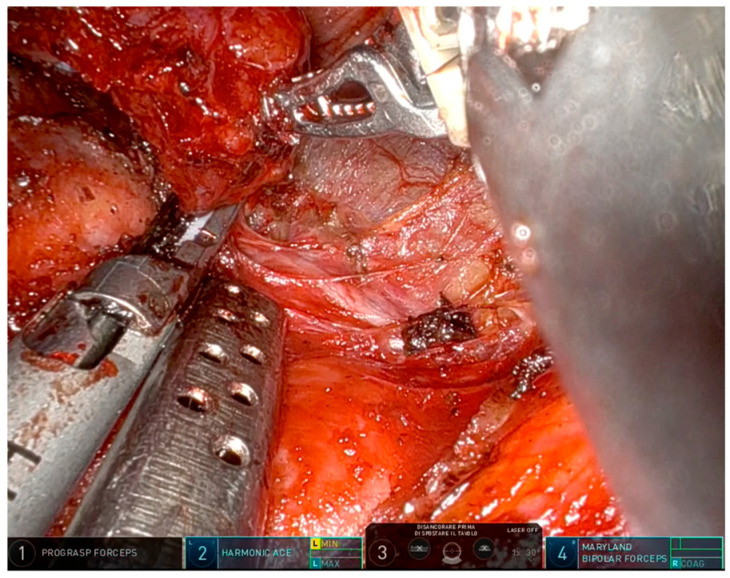
Robotic arm configuration in left inferior thyroid lobe dissection.

Once the RLN is identified and confirmed via IONM, it is carefully dissected free along its entire course to its point of tracheal insertion. To enhance visualization of the nerve’s full trajectory, the “swing technique” is employed: the 30° robotic endoscope is repositioned from arm 2 to arm 3, and, if necessary, to arm 4, before being returned to arm 2 at the end of the procedure (Figure 6).

This optical movement strategy is developed when using the Xi system (Intuitive Surgical Inc., Sunnyvale, CA, USA). In fact, with the introduction of the Da Vinci Xi system, the 5 mm thin robotic instruments used with the previous system have been replaced by more agile but bulky 8 mm instruments. To overcome a possible increase in steric interference and the possibility of collision between instruments within the confined operating space of the TA working space, we therefore decided to move the optics to a different position on the arms in order to follow the course of the RLN throughout the entire procedure. In other words, this approach improves visualization within the limited operative space, clarifies the anatomical course of the RLN and contralateral parathyroid glands, and facilitates precise and meticulous dissection, thereby minimizing the risk of RLN injury or residual thyroid tissue. This technique maximizes the advantages of the image magnification capabilities offered by the robotic system, minimizing the risk of incomplete surgical removal of the contralateral thyroid lobe. The advantages of the swing technique could also be effective with the use of Da Vinci 5.

The remaining thyroid lobe is then completely excised using the Ultracision© dissector. At the conclusion of the procedure, meticulous hemostasis is verified, and a Valsalva maneuver is performed to detect any occult venous bleeding. During this maneuver, partial release of the external retractor is recommended to allow optimal visualization of the subcutaneous tunnel.

A drain is placed in the thyroid bed and exteriorized through the most caudal trocar incision, typically removed within 24 h. The axillary incision is finally closed.

## 3. Discussion

Gasless transaxillary robotic thyroidectomy (G-TART) demonstrates surgical outcomes comparable to conventional open thyroidectomy (OT) in terms of safety, complication rates, and oncologic completeness, with the added benefits of superior cosmetic satisfaction [3,6,7,8].

Traditionally, there are several factors that have limited the widespread use of the technique: high cost, long operative time and difficult learning curve.

However, operative times have improved with technological advancements, notably with the da Vinci Xi (Intuitive Surgical, Sunnyvale, CA, USA) [9] and SP system (Intuitive Surgical, Sunnyvale, CA, USA) [10], without increasing complications. Although evidence is predominantly from retrospective cohorts and single-center series, the current literature evidence consistently supports TART as a feasible, safe, and cosmetically advantageous alternative for selected patients with thyroid disease, particularly with benign histology or papillary thyroid carcinoma.

TART is offered, in accordance with ATA guidelines [11], recent European recommendations [12] and Italian consensus statement [13], for highly motivated patients with aesthetic concerns and the presence of small (<2 cm), benign, unilateral thyroid nodules, in the context of a thyroid lobe measuring no more than 6 cm in maximum diameter.

The implementation of these recommendations has, over time, become a necessity driven by the introduction of the transaxillary robotic technique in European and American populations, which exhibited characteristics different from those of the reference Asian population.

In high-volume centers specializing in both endocrine and robotic surgery, where the robotic approach to thyroid pathology is being newly introduced, initial candidates are typically young, skinny, euthyroid individuals without comorbidities and with small benign nodules. As surgical expertise progresses, indications may be expanded to include patients with larger nodules and those diagnosed with low- to intermediate-risk papillary thyroid carcinoma. Carefully selected patients with Graves’ disease may also be considered suitable candidates for a robotic transaxillary approach.

Absolute contraindications include advanced thyroid cancer with extracapsular extension, or gross lymph node metastasis; substernal goiters; a history of cervical surgery or prior irradiation to the head and neck; and the presence of cardiac pacemakers.

Several studies, such as that by Papini et al. [8], confirm the efficacy and safety of this approach also in the European population. The Pisa group analyzed the outcomes of 242 patients with a histological diagnosis of thyroid cancer over an eight-year period (2012–2020), of whom 47% underwent robotic transaxillary lobectomy. Twenty-eight patients subsequently underwent a complete lobectomy. Reported complication rates included 1.2% transient laryngeal nerve palsy, 0.8% transient hypoparathyroidism, and 0.8% hematoma. Furthermore, at a median follow-up of 38 months, the authors reported that 37% of patients underwent postoperative radioactive iodine ablation therapy. The authors also concluded that there was no evidence of local recurrence or distant metastases in the entire cohort, with an excellent response to therapy observed in 74% of patients.

The body of literature on G-TART has significantly matured over the past decade, with numerous retrospective analyses [8] and comparative studies [6] elucidating its surgical outcomes. Recently, Kim et al. reported the largest clinical experience to date with gasless transaxillary robotic thyroid surgery, encompassing 10,000 patients at the Yonsei University Health System in Seoul, Korea. They documented postoperative complication rates of 37.91% for transient hypocalcemia and 2.1% for transient recurrent laryngeal nerve palsy, while the rates for permanent hypocalcemia and RLN injury were 1.52% and 0.32%, respectively. These outcomes are comparable to those reported for conventional OT, supporting the safety and efficacy of TART as an alternative approach. Furthermore, the authors detailed the progressive expansion of surgical indications over time. With increasing surgical proficiency, the robotic TA approach was safely extended to patients with large goiters, advanced thyroid cancer—due to the capacity to perform precise central compartment lymph node dissections—muscular male patients, and individuals with cancer recurrence. The authors suggest that the TA approach offers a surgical field and visualization comparable to that of conventional OT. Notably, reoperation for postoperative bleeding or recurrent disease is now considered safe and feasible via the TA route [14].

Several comparative studies have also supported the favorable outcomes associated with TART. Notably, Xing et al. [15] in a network meta-analysis including 30 studies and a total of 6622 patients, found no significant difference in the number of central compartment lymph nodes retrieved when comparing TART with the conventional OT. However, in pairwise meta-analysis, the TA technique was associated with a higher number of metastatic lymph nodes retrieved compared to OT. The authors also reported that the TA approach required less operative time than both the bilateral axillo-breast approach (BABA) and the transoral (TO) approach. In terms of cosmetic outcomes, TA thyroidectomy demonstrated significantly greater patient satisfaction compared to the OT. Regarding postoperative complications, TART showed no statistically significant differences in rates of transient hypocalcemia and yielded comparable results in terms of RLN palsy.

Given the well-documented advantages and well-established outcomes of TART, our surgical team has consistently pursued further refinement of the technique with the aim of potentially enhancing its safety profile. A significant challenge associated with the TA approach, particularly when accessing the contralateral thyroid lobe, is the limited operative field and restricted visualization, which complicate the safe identification and preservation of the RLN. To address these limitations, we implemented the “swing technique”, a dynamic endoscopic port-repositioning maneuver, in combination with a specialized intraoperative neuromonitoring (IONM) probe (Modena Robotic Probe). This modification significantly improved anatomical exposure, allowing for more accurate RLN mapping and preservation. The swing maneuver was further optimized by integrating a concave-shaped IONM probe, specifically engineered for use in confined surgical fields, which enhanced neural monitoring capabilities during the procedure. The technical synergy of these innovations may have contributed to more reliable identification of the recurrent laryngeal nerve and to a significant increase in the precision and accuracy of surgical maneuvers. These adjustments are particularly advantageous when using the Da Vinci Xi system (Intuitive Surgical, Sunnyvale, CA, USA), and they remain fully compatible with the Da Vinci 5 system (Intuitive Surgical, Sunnyvale, CA, USA). Importantly, the use of a specially designed IONM probe remains essential across all robotic approaches to the thyroid gland. Its ergonomic concave shape permits greater control and precision by the bed-side assistant surgeon, contributing to safer and more effective nerve monitoring.

## 4. Conclusions

Thanks to our consolidated experience in gasless transaxillary robotic thyroidectomy, the integration of the swing technique, a dynamic camera repositioning across the robotic arms, together with a dedicated intraoperative nerve monitoring probe specifically engineered for robotic use, represents a meaningful technical advancement. This combined approach may further enhance intraoperative visualization and is likely to facilitate more reliable identification and preservation of the recurrent laryngeal nerve, while also allowing for more precise dissection within the confined operative field characteristic of the transaxillary approach. Such technical refinements address intrinsic limitations of robotic surgery and may contribute to improved surgical outcomes and a reduction in complication rates. Further studies are warranted to evaluate reproducibility and long-term safety, with the potential for this methodology to be established as a standard technique in transaxillary robotic thyroidectomy.

## Figures and Tables

**Table 1:** Surgical equipment.

Hybrid phase: working space Modena Retractor (CEATEC^®^ Medizintechnik GmbH Wurmlingen, Germany) equipped with valves of varying widths and lengths; Suction device with a fine cannula (Modena Retractor—(CEATEC^®^ Medizintechnik GmbH Wurmlingen, Germany)); Suction tubing connected to the valve mounted on the Modena Retractor (CEATEC^®^ Medizintechnik GmbH Wurmlingen, Germany); Radiopaque gauze pads, 25 × 25 cm; Standard scalpel handle No. 3 with mounted scalpel blade No. 10 for incision; Two standard surgical forceps, 18 cm; Two Farabeuf retractors; Kelly clamp, 24 cm, with mounted 12 mm radiopaque cotton swab; 30° angled laparoscopic camera; Monopolar electrocautery with short and long tip extension; Laparoscopic hook (crochet) for dissection and subcutaneous space creation; Laparoscopic bipolar forceps for hemostasis; Johannes clamp; Optical warming thermos; Narrow standard anatomical forceps, 30 cm, used as a guiding valve to facilitate advancement of the retractor valve during subcutaneous tunneling; Scissors, 30 cm; Thin malleable retractor, 25 cm.
Robotic phase The Da Vinci Xi system (Intuitive Surgical Inc., Sunnyvale, CA, USA) Robotic ProGrasp forceps, 8 mm; Ultracision© Ethicon Inc, Raritan, NJ, USA robotic dissector, 8 mm; Robotic Maryland bipolar forceps, 8 mm; 30° angled endoscope, 8 mm; Modena Robotic Probe (Model 40-0038, Dr. Langer Medical GmbH, Waldkirch, Germany), AVALANCHE^®^ SI2 neuromonitor (Dr. Langer Medical GmbH).

## Data Availability

No new data were created or analyzed in this study.

## Abbreviations

The following abbreviations are used in this manuscript:

G-TART	Gasless transaxillary Robotic Thyroidectomy
IONM	Intraoperative Nerve Monitoring
RLN	Recurrent laryngeal nerve
TA	Trans axillary
OT	Open thyroidectomy
TART	Trans axillary robotic thyroidectomy
SCM	Sternocleidomastoid muscle
PTFE	Polytetrafluoroethylene
BABA	Bilateral axillo-breast approach
TO	Transoral
